# Systems thinking in, and for, public health: a call for a broader path

**DOI:** 10.1093/heapro/daae086

**Published:** 2024-08-13

**Authors:** Erica Wirrmann Gadsby, Helen Wilding

**Affiliations:** Faculty of Health Sciences and Sport, University of Stirling, Stirling FK9 4LA, UK; Applied Systems Thinking in Practice group, The Open University, Walton Hall, Milton Keynes MK7 6AA, UK

**Keywords:** systems, systemic, inequalities, transformation, partnership, empowerment

## Abstract

Systems thinking has been recognized as valuable to public health policy, research and practice. Commentators and reviews have highlighted that there is still much to be done to embrace its potential. Here, we highlight that much of the discourse about systems thinking in, and for, public health supports the pursuit of a narrow path and is limited with respect to the lineages of Systems that are embraced. We invite readers to see the potential of systems thinking in pursuing a broader path which is motivated by a concern for alleviating health inequalities. This does not replace the narrow path but encompasses it. It prompts different considerations with respect to the nature of the transformation, partnership working and legitimacy. It also invites a different way of engaging with systems thinking and different ways of conceptualizing and managing change. The broad path both requires, and helps enhance, new ways of doing, relating, organizing, knowing and framing which are vital for the future of public health as a global concern.

**Contribution to Health Promotion**

- Systems thinking has the potential to enhance health promotion.
- However, there is a risk that it is deployed in a limited narrow way with a focus on enhancing disease reduction approaches to public health.
- We describe a broader path which differs with respect to the nature of the transformation, partnership working, use of Systems ideas and tools, nature of legitimacy and underpinning understandings of change.

## INTRODUCTION

A strong case has been made for public health policy, research and practice to embrace systems thinking [e.g. (Midgley, 2006; Leischow *et al*., 2008; Peters, 2014; Russell *et al*., 2014; Rutter *et al*., 2017; Haynes *et al*., 2020; Hostford, 2020; Kavanagh *et al*., 2020; Lauwerier *et al*., 2021; World Health Organization European Region, 2022)]. This reflects the complexity of public health challenges, the need to focus on underlying causes and the importance of an integrated, collaborative approach. There is a growing sense that those working in and for public health must incorporate systems thinking into their practice to improve population health and reduce health inequalities.

Systems thinking is often seen as vague, abstract and confusing. It is described as a core skill or competency in public health and health promotion (Rocheleau *et al*., 2022; Paina and Glenn, 2023; Public Health Network Cymru, 2023), and as an approach to problem-solving or to dealing with complex struggles more effectively [e.g. (de Savigny and Adam, 2009; Morgan *et al*., 2023; Thelen *et al*., 2023)]. It is also described variously as a discipline (Swiss Tropical and Public Health Institute, 2023), a framework (Knai *et al*., 2018; Government Office for Science, 2023), a conceptual rubric (Leischow *et al*., 2008), an area of knowledge (World Health Organization European Region, 2022), an ‘ability’ (Dolansky *et al*., 2020) and a set of principles (McNab *et al*., 2020). It is notable that these explanations emphasize *individual* capabilities.

Many frameworks have been developed to explain systems thinking in public health [e.g. (Best *et al*., 2003; Kapp *et al*., 2017; Bolton *et al*., 2022; Card, 2022; Thelen *et al*., 2023; Smith *et al*., 2024; NHS North West Leadership Academy, n.d.)]. These frameworks often resemble other holistic approaches like One Health (Ghai *et al*., 2022), EcoHealth (Charron, 2012), Planetary Health (Iyer *et al*., 2021) and Health in All Policies (World Health Organization & Finland. Ministry of Social Affairs and Health, 2014). They emphasize the need to focus on upstream determinants of health and to collaborate across different sectors and stakeholders. Here, systems thinking or ‘whole systems’ working becomes synonymous with intersectoral collaboration and partnerships, shifting the focus *away* from the practice of individuals.

These frameworks both manifest and promote more systemic approaches to health. However, using the language of systems thinking does not always mean that it is taking place. It has been observed that there are critical shortcomings in what is used and how (Carey *et al*., 2015; Chughtai and Blanchet, 2017) and its impact on public health policy makers and practitioners in the field remains limited (Boswell *et al*., 2021). This might be partly because, as Carey *et al*. point out (Carey *et al*., 2015), people working in and for public health are engaging with only a limited range of systems methodologies, barely tapping into its potential. We have noticed a tendency to adopt the terminology of systems thinking without really thinking (or acting) differently, or delivering different outcomes, to so-called ‘traditional’ approaches. As Chughtai and Blanchet (Chughtai and Blanchet, 2017) noted in conclusion to their review of systems thinking in public health, there is a need for greater interdisciplinarity and a willingness to engage with unfamiliar methods and combinations. We also observe that work to support place-based approaches, such as Public Health England guidance (Public Health England, 2021) or The Health Foundation’s ‘Shaping Places’ programme (The Health Foundation, 2022), talks in terms of ‘local systems’ but does not explicitly promote the use of Systems ideas and approaches.

In recent years, efforts have been made to promote the application of systems thinking in public health research. For instance, the UK Academy of Medical Sciences emphasized the need for research to understand the complexities of interactions within adaptive systems, stressing the importance of transdisciplinary and innovative approaches (Academy of Medical Sciences, 2016). Subsequently, the UK Prevention Research Partnership was established in 2017 to fund innovative research on non-communicable disease prevention, focused on solutions, policies and strategies within complex adaptive systems. To date, the partnership has funded seven large interdisciplinary research consortia to tackle specific challenges.

In public health practice, there have been several national-level initiatives to promote systems thinking in addressing complex public health issues. In England, a significant focus has been on tackling obesity, with Public Health England investing in resources to guide local authorities in implementing whole systems approaches to obesity (Public Health England, 2019b). In Australia, the Australian Prevention Partnership Centre has been a champion for promoting systems thinking in chronic disease prevention since 2013 and has applied systems thinking to various areas such as food supply systems, prevention financing, monitoring and evaluation systems and prevention regulation and legal systems. They have also ‘applied systems science to identify new solutions for obesity, food insecurity, diabetes, physical inactivity, alcohol consumption’ and so on (The Australian Prevention Partnership Centre, 2023, p. 13). More broadly, the English level seven Systems Thinking Practitioner apprenticeship standard is recommended as one which supports public health careers (Public Health England, 2019a), and the UK Government Office for Science has produced a suite of practice-oriented documents, including a toolkit, to assist civil servants in integrating systems thinking into their work (Government Office for Science, 2022).

In late 2020, an international expert group, convened by the UK Academy of Medical Sciences and the Canadian Academy of Health Sciences, held online workshops to discuss systems-based approaches in public health and their advancement. The report summarizing that work, entitled ‘Systems-based approaches in public health: Where next?’ served as a catalyst for this commentary (Jebb *et al*., 2021). As systems thinking practitioners from different disciplines with experience in public health, we noted the expert group’s call for methodological innovation to advance the field and to deepen the understanding and application of systems-based approaches in public health research and practice. We are responding to this call in further considering the breadth of potential that practice, drawing on Systems ideas, offers to the future of public health.

## THE ART AND SCIENCE OF PUBLIC HEALTH PRACTICE

Reflecting on the future of public health, we start with a metaphor articulated by Beaglehole and Bonita 20 years ago (Beaglehole and Bonita, 2004). Their metaphor of a crossroads suggested two possible directions for public health: a broad one aimed at reducing health inequalities and promoting overall well-being and a narrow one aimed at reducing individual risks to reduce disease prevalence. Hahn (Hahn, 2019) similarly discussed two alternative, divergent paths to Health in All Policies: traditional public health and the path of social determinants. While these distinctions are helpful, we believe the crossroads metaphor implies a dualism and suggests a false choice between paths. Instead, we see the narrow path as part of the broader one, forming a duality where the two elements form a whole.

Experience has shown that following the broad path in public health is challenging. Despite good intentions, the focus often narrows as the journey progresses—akin to wearing blinkers that screen out distractions and limit one’s perspective. This is understandable, as social-structural perspectives are harder to grasp compared to physical or psychological explanations [e.g. (Walsh *et al*., 1995)]. Western societies tend to attribute social issues to personal traits rather than systemic factors, favouring reductionism. Acknowledging the significance of social structures in health can feel overwhelming, leading to a sense of powerlessness (Wirrmann, 2004). However, this narrowing perspective risks failing to address global health threats. The term ‘lifestyle drift’ describes this tendency, where efforts initially acknowledge social, economic, political and commercial determinants of ill-health and inequalities, but regress to designing policies targeted largely at modifying the behaviours of individuals (Popay *et al*., 2010).

In this paper, we consider the broad and narrow directions for public health articulated by Beaglehole and Bonita, viewing them as a duality, rather than dualism. We also reflect on how public health practitioners have approached systems thinking, identifying both broad and narrow paths for systems thinking in, and for, public health. We propose that a more comprehensive embrace of systems thinking could open up broader possibilities for public health. Using our metaphor, we encourage practitioners to remove the blinkers, and instead to don eyewear that offers a kaleidoscope of perspectives drawn from the rich and multi-disciplinary lineages of Systems. Here, we define practitioners as all those directly and indirectly involved in public health practice, research and policy in the pursuit of population health improvement.

Below, we begin by outlining different ways of engaging with systems thinking, in order to more fully introduce the broad and narrow paths for systems thinking in, and for, public health.

## WAYS OF ENGAGING WITH SYSTEMS THINKING

Many people naturally think in terms of relationships. It has been argued that individuals possess this sensibility from birth but may not always retain it as they grow up (Ison and Straw, 2020). However, this sensibility can be enhanced by purposefully drawing on ideas and approaches from the field of Systems, whether practising alone or collaboratively [we follow Ison (Ison, 2017) by capitalizing the ‘S’ when we refer to the academic area of study underpinning systems thinking].

Practice that is informed by Systems is particularly valuable when working in, and acting to improve, situations experienced as complex, messy or contentious because the ideas and approaches assist in understanding relationships between entities, engaging with multiple perspectives, and navigating power dynamics and conflicts (Reynolds and Holwell, 2020a). Different terms have been coined to refer to situations experienced this way, such as wicked problem (Rittel and Webber, 1973), mess (Ackoff, 1974), swamp (Schön, 1991) and problematical situation (Checkland, 1999). Furthermore, frameworks have been advanced for helping make distinctions between types of situation. As examples, the ‘system of systems methodologies’ makes distinctions based on the degree of interrelatedness in the situation and degree of conflict between stakeholders (Jackson and Keys, 1984); Stacey’s agreement and certainty matrix is based on continua related to certainty/uncertainty and agreement/disagreement (Zimmerman, 2014); and the CYNEFIN framework distinguishes between disorder, obvious, complicated, complex and chaotic situations (The Cynefin Co, 2024). These terms and frameworks were devised in order to make sense of, and communicate, experience. However, where the terminology is used as fixed categories, there is an assumption that everyone is experiencing the situation in the same way (Checkland, 1999). It can also mean the potential for learning in context is overlooked, since ‘we can become trapped in particular ways of engaging with situations’ (Ison, 2017, p. 133).

Systems, like other fields, is not a homogenous area of study; its scope and nature can be contested (Ison, 2017). Influential thinkers from many disciplines have shaped its development, including those associated with general systems theory, cybernetics, complexity theory, soft and critical systems, and learning systems (Ison, 2017; Ramage and Shipp, 2020). These frameworks of ideas and approaches have been applied, and further shaped, in the study and improvement of natural, mechanical, social and human activity systems. This has led to several recognized systems approaches, including system dynamics, viable system model, strategic options development and analysis, soft systems methodology and critical systems heuristics (Reynolds and Holwell, 2020b). However, within public health research and practice, published literature suggests that it is systems dynamics, with its associated methods of causal loop diagramming and group model building, that has gained most traction.

The different lineages of Systems open a spectrum of standpoints with respect to whether the term ‘system’ is understood, and used, from an ontological standpoint or an epistemological one (Ison, 2017; Reynolds and Holwell, 2020a). At the ontological extreme, a system is understood to be out there in the world waiting to be understood and then engineered so that it performs in a more desirable way. The alternative is to adopt a stance which uses systems as constructs or devices (epistemologies) to help in understanding, and acting to improve messy, problematic situations. This distinction is at the heart of a contrast between systematic thinking and systemic thinking, which are compared in Table 1.

**Table 1:** A comparison of systematic thinking and systemic thinking [adapted from (Checkland, 1985, p. 765) and (Ison, 2017, p. 160)]

	Systematic thinking	Systemic thinking
Checkland’s terminology (Checkland, 1985)	Hard systems thinking	Soft systems thinking
Useful when	Problems need solutions.	Issues require accommodation. Need to keep in touch with human content in a situation where linear logic may not apply.
Orientation	Goal seeking.	Learning.
Assumption	World contains systems which can be engineered.	World is problematical.
Role of system models	They model the world (ontologies). Their development often depends on the use of powerful techniques.	They are intellectual constructs that help the modeller(s) understand their own, and others’, perspective of the world (epistemologies).
Ends when	The right answer is identified.	No final answers, inquiry never ends.

Here, once again, we urge readers to view this pair as a duality that together make systems thinking. Ison (Ison, 2017, p. 196) highlights that systemic thinking provides an ‘expanded context’ for systematic thinking. Similarly, Checkland (Checkland, 1985, p. 766) refers to soft systems thinking as ‘the general case of which “hard” systems thinking is the occasional special case’. In other words, systemic thinking can be thought of as a broader path which encompasses, rather than rejects, the appropriate use of narrower systematic thinking. We use the term ‘systems thinking’ to refer to the duality—both systemic and systematic.

We emphasize that there is nothing inherently ‘systematic’ or ‘systemic’ about individual ideas or approaches from the field of Systems. The distinction serves to provide a choice about how to engage with systems thinking, not to categorize tools and ideas. Increasingly, systems practitioners agree that systems are conceptual constructs (Reynolds and Holwell, 2020a) and report that it is more appropriate to start out by engaging systemically with a situation (Ison, 2017).

## PATHS FOR SYSTEMS THINKING IN, AND FOR, PUBLIC HEALTH

We consider Beaglehole and Bonita’s distinction between the broad and narrow paths to be manifestations of the two different ways of engaging with systems thinking. Systematic thinking comes to the fore in the narrow path in the way that it emphasizes powerful epidemiological techniques and shorter-term risk reduction. The broader path shows evidence of systemic thinking by incorporating opportunities to appreciate perspectives through more participatory methods and striving towards long-term global benefits.

Thus, the paths invite us to engage with systems thinking differently. We see two different possibilities building on Beaglehole and Bonita’s articulation of the motivating concerns within each path. We express these paths using a structure that systems practitioners deploy to define a human activity system by focusing on the What? How? and Why? (Armson, 2011, p. 215):

*Narrow:* To (what) reduce risk of disease by means of (how) research, policy and practice enhanced with systems thinking and action in order to (why) bring about the absence of disease.

*Broad:* To (what) alleviate inequalities in health by means of (how) research, policy and practice enhanced with systems thinking and action in order to (why) bring about a state of complete physical, mental and social well-being.

Below, we consider different ways that these narrow and broad paths compare.

### The nature of the transformation

The narrow and broad paths contrast in terms of the desired transformation. As highlighted above, the core purpose of a narrow path is to reduce risk of disease. It seeks to benefit sub-sets of a population who are at risk of developing disease, such as smokers or those who are overweight or obese, for example. Success is determined using measures associated with the proportion of populations with risky behaviours. In contrast, the beneficiaries of a broad path are taken to be current and future society, whether understood to be at local, national or global level. Success is understood in terms of the presence of physical and socio-structural environments that are conducive to good health and positive well-being for all. The broader path encourages a ‘whole of health’ approach (Wilding, 2021, p. 24) that focuses on different upstream determinants irrespective of the specific outcomes ‘downstream’. In Lang and Rayner’s words (Lang and Rayner, 2012, p. 2), this requires ‘complex ecological thinking’.

As Katikireddi *et al*. (Katikireddi *et al*., 2013) observe, public health research, policy and practice tend to be organized to focus on particular health issues or behaviours. Health and well-being are not worked with holistically but disaggregated and reduced into often silo-ed areas of activities associated with particular measures of improvement. So, instead of a determinants based, whole of health approach, recommendations and action are centred on ‘a whole systems approach to’, for example, obesity (Public Health England, 2019b), childhood tooth decay (Local Government Association, 2019), physical activity (Nau *et al*., 2022) or mental well-being (Cefai *et al*., 2021). There is, as Lang and Raynor (Lang and Raynor, 2012) highlight, a diminution of perspective arising from a view of public health as a set of interventions or a set of laws or technologies led by professional expertise (often targeted at those with higher risk of disease). This has discouraged attention on the big picture and the social-structural forces that shape people’s health and well-being.

### The nature of partnership working

The narrow and broad paths contrast in terms of who is involved, and how. Both paths invite a so-called ‘whole systems approach’ in that there are expectations for the involvement of a variety of decision makers who command the use of relevant resources and policy levers. The idea of partnership working for health is not new. As far back as the Alma-Ata Declaration (International Conference on Primary Health Care, 1978), there has been a concern for both community participation and the governance and working arrangements that link health horizontally with other policy sectors (Kickbusch and Gleicher, 2012). However, there is a great variety of partnership working, both in theory and in practice, and these very rarely involve the authentic reallocation of power understood to be required for meaningful participation (Arnstein, 1969).

Seminal UK work on ‘whole systems working’ led to the development of a typology of different forms of partnership working based on whether goals are individual or collective and whether predictability is high or low (Pratt *et al*., 1998, 1999; Gordon *et al*., 2010; Pratt and Plamping, 2010). In the narrow path, epidemiologists and the core public health workforce seek to establish high predictability about what needs to be done to reduce the risk of disease. A collective goal is assumed, and others are invited to play their role. This fits with the pattern that Gordon *et al*. (Gordon *et al*., 2010) refer to as coordination, accompanied by the image of a jigsaw to represent the idea that, if each partner contributes, the picture is more complete.

The broad path involves taking what Kickbusch and Gleicher (Kickbusch and Gleicher, 2012) refer to as a whole of government and whole of society approach. But as the path broadens, and the nature of health and well-being are contested, it is increasingly difficult to agree a collective goal, and there is less certainty about what works. This invites a co-evolving form of partnership where those involved explore together, share perspectives, iterate, learn and over time take responsibility for the ‘whole’, rather than individual contributions.

### The role of ideas and tools from Systems

The two paths differ with respect to how practitioners engage with Systems. In the narrow path, systems thinking in public health is framed predominantly as a tool to avoid the trap of reductionism. Systems approaches such as system dynamics and the concept of a complex adaptive system are promoted to advance knowledge of the determinants of disease. In this context, experts use sophisticated epidemiological techniques enhanced by systems modelling to produce knowledge that helps people to understand, and subsequently engineer, systems (as ontological things). A great many causal loop diagrams have been produced in recent years, for instance, to understand complex issues such as obesity, mental health and opioid use. However, as others have pointed out, the use of systems tools does not necessarily challenge traditional reductionist epistemologies (Burns, 2007; Reynolds *et al*., 2018; Riley *et al*., 2021).

In the broad path, there is greater recognition of the need to appreciate multiple perspectives to avoid the trap of dogmatism. To some extent, this can be achieved through pluralistic approaches in research, for example using a salutogenic model as well as a pathogenic one; drawing on multiple models of public health, such as those outlined by Lang and Rayner (Lang and Rayner, 2012); and, ensuring the contribution of a variety of research disciplines such as medicine, psychology, economics, social and political sciences, health services research, humanities, geography and legal science (Kivits *et al*., 2019).

However, the broad path goes further to invite practitioners to recognize multiple ways of knowing. This dissipates the distinction between knowledge ‘producers’ and knowledge ‘users’ and invites us to accept Cook and Wagenaar’s (Cook and Wagenaar, 2012) proposition that there is a dynamic integration of knowledge, practice and context. In this context, the offer of Systems traditions is less to do with their use as methods for the initial investigation of the ‘problem’ and more to do with the way in which they enable people working alone or collectively to understand interrelationships, appreciate other perspectives, reflect on boundary judgements and take desirable, feasible and ethically defensible actions in pursuit of better health and well-being for all.

It is recognized that not everyone will have the opportunity to formally develop their knowledge and ability to use systems approaches. However, in a partnership context, it is possible for a skilled facilitator to use ideas and tools arising from a variety of Systems traditions in bricolage with participatory approaches, such as Open Space or World Café, to create the circumstances where systemic sensibilities are expressed and nurtured. Working in this way requires skills and attitudes that have been associated with competent boundary spanners (Williams, 2013) and systems convenors (Wenger-Trayner *et al*., 2015; Wenger-Trayner and Wenger-Trayner, 2021).

### The nature of legitimacy

Both the narrow and broad paths include some stakeholders and exclude others. It is important to critically reflect on whose voices are privileged, whose are not involved and what should be done about emancipation (Ulrich and Reynolds, 2020).

The narrow path focuses on specific concerns that are usually identified as a priority through data or performance measures. This privileges the professional judgement of public health practitioners and sometimes political involvement in priority setting. The perceived beneficiaries of interventions, such as those who are overweight, those who smoke or those who live in a certain low-income neighbourhood, have little voice in setting this priority. If given the opportunity, members of a community may identify very different concerns that they perceive have a negative impact on health and well-being. In some cases, the primary response to these concerns may not be with public health practitioners, but with other sectors such as policing, parks or street cleaning.

The broader path is much more diffuse. A greater range of stakeholders may become involved, and any one individual will have multiple stakes as beneficiaries, decision makers and contributors of experience and expertise. However, there is still a boundary, and it must be recognized that there are some without voice, such as future generations and the non-human biosphere. In an inter-connected, global economy, there are also distant stakeholders who are affected by, but unable to be involved in, local-level public health action. It is important to recognize that even the broad path risks health imperialism, where it privileges the view that health should be the primary interest of all involved. There may be times when it is important to recognize the inter-linkages of societal challenges and join others on a path that is ‘for well-being’, ‘for equity’ or ‘for sustainability’ rather than primarily ‘for health’.

### The nature of change

Our final point of reflection is associated with how each path reflects different perspectives of what change is and what is done to ‘manage’ it. In the narrow path, the tendency is to privilege a blueprint view. This is a mode of thinking that assumes that change can be planned (often by experts, specialists or professionals) and then implemented (Vermaak and de Caluwé, 2018). This can be seen, for example, in systems approaches to obesity adopted by many local public health teams in the UK. A variety of people are brought together to apply systems tools and methods to better understand what ‘drives’ obesity in their local area, and to identify interventions. Such work can be valuable in building shared commitments and enhanced understanding, and can result in people doing things differently, alone or together. However, they assume that when you implement the structure of a ‘whole systems approach’, you will achieve desired outcomes. Sometimes, in a strive towards methodological rigour and robustness, the narrow path can call for quite prescriptive following of a specific approach, often to be implemented in a structured, step-by-step way.

Our broad path invites us to consider change as a gradual co-evolution, rather than a before-after implementation. This sort of change requires dialogue and self-organization, negotiation, learning and development, and emergent solutions. Here, one can be informed by different ways of thinking about change. For example, Vermaak and de Caluwé have proposed a colours of change framework that offers a nuanced view of change, recognizing different belief systems and convictions about how change occurs (Vermaak and de Caluwé, 2018). The authors emphasize the importance of context and the need for change agents to be adaptable, utilizing a combination of approaches where necessary. The framework encourages flexibility, reflection and the strategic use of multiple approaches to foster successful change initiatives.

Given the entrenched and complex social, political and economic determinants of health, the change required must be transformative and systemic at both an individual and collective level. A number of ideas have been advanced with respect to how to understand and facilitate collective systemic change. Many of these are discussed in Blackmore’s (2010) edited volume on social learning systems and communities of practice which brings together important contributions from Donald Schön, Geoffrey Vickers, Richard Bawden and Etienne Wenger.

There is also much to be learned from a recent international research project (see www.transitsocialinnovation.eu) which set out to understand processes of societal transformation. Within this work, Haxeltine *et al*. highlight that change is transformative when it leads to new ways of doing, relating, organizing, knowing and framing (Haxeltine *et al*., 2016). This requires extensive reflection on ‘what do we do when we do what we do?’ (Ison, 2017, p. 5).

## CONTINUING OUR JOURNEY

The report that acted as a catalyst for this article posed the question ‘Where next?’ for systems-based approaches in public health (Jebb *et al*., 2021). Recognizing that the ‘systematic application’ of systems-based approaches in public health remains the exception rather than the rule, the Expert Group set up to investigate the issue suggested that the answer to the ‘Where next?’ question lies in: generating and synthesizing evidence of added value; developing a community of practice to share evidence, support and promote new and existing approaches; and target funding for systems-based approaches and for capacity building. We agree that these actions would be helpful, but our analysis of the situation suggests that these actions alone may help us pursue the narrow path better, to the exclusion of fully embracing systemic thinking that can enable the pursuit of the broad path. We suggest that our broad path entails:

- (In relation to motivation) maintaining a desire to make meaningful progress towards population health improvement underpinned by a broad perspective of health and well-being.
- (In relation to partnerships) valuing co-evolution as a collective enterprise, where understandings and practices of all those involved (including the so-called experts) are open to change.
- (In relation to systems ideas and tools) drawing on a broader range of Systems lineages and contemporary systems thinking tools and methods than at present and recognizing multiple ways of knowing.
- (In relation to legitimacy) being aware of, and reflecting on, our inevitable boundary judgements and the potential traps of health imperialism.
- (In relation to change) opening up to a wider range of views of what change is and how it happens.

Ultimately systems thinking in, and for, public health has the potential to be transformative, both in terms of innovations in public health practice and in terms of public health outcomes. However, this will entail embracing the richness of Systems more fully, to engage in and bring about new ways of doing, relating, organizing, knowing and framing. This is a journey that will both require and lead to changes at the level of institutions and social structures (for example, in relation to governing, funding, science, methodology, publication and education). We hope that this paper, and the systems thinking traditions we have drawn on, opens up possibilities and contributes to the ongoing dialogue in this journey.

## Data Availability

No new data were generated or analysed in support of this research.
